# Machine learning-based prediction of acute and complicated appendicitis using readily available data in low-resource settings

**DOI:** 10.1371/journal.pone.0339299

**Published:** 2026-02-03

**Authors:** Kimya Bahamin, Arya Derakhshesh, Mohammad Hossein Mahmoudi, Omid Kohandel Gargari, Mohammad Ali Takhshid, Mehdi Amirfazli, Arash Dehghan

**Affiliations:** 1 Student Research Committee, Hamadan University of Medical Sciences, Hamadan, Iran; 2 Industrial Engineering Department, Sharif University of Technology, Tehran, Iran; 3 Student Research Committee, Alborz University of Medical Sciences, Karaj, Iran; 4 Department of Laboratory Sciences, School of Paramedical Sciences, Shiraz University of Medical Sciences, Shiraz, Iran; 5 School of Biological Sciences, Illinois State University, Normal, Illinois, United States of America; 6 Department of Pathology, School of Medicine, Hamadan University of Medical Sciences, Hamadan, Iran; Faculty of Medicine Vajira Hospital, Navamindradhiraj University, THAILAND

## Abstract

**Background:**

Acute appendicitis often presents diagnostic challenges, especially in pediatric and elderly patients. Delays can cause serious complications. While imaging aids diagnosis, it is not always accessible. Machine learning offers a promising solution. This study aimed to develop a simple, accurate model using basic demographic and laboratory data to improve diagnosis in low-resource settings.

**Materials and methods:**

This retrospective, single-center study analyzed 453 patients undergoing appendectomy for suspected acute appendicitis. Clinical, laboratory, and histopathological data were collected and classified into normal, uncomplicated, or complicated appendicitis. Data preprocessing included feature encoding, scaling, and balancing. Seven machine learning models were trained and evaluated using stratified five-fold cross-validation, and interpretability was assessed using SHapley Additive exPlanations (SHAP).

**Results:**

Among 453 patients, appendicitis was confirmed in 68.87%, predominantly in males (p < 0.001). Patients with appendicitis were significantly older (p = 0.0012), exhibiting elevated white blood cells(WBC) count, neutrophils, and C-reactive protein (CRP) levels, and lower lymphocytes (all p < 0.001). The Support Vector Classifier (SVC) performed best in classifying appendicitis (accuracy = 75.82%, ROC-AUC = 76.39%). SHAP analysis identified WBC, lymphocyte percentage, gender, age, and neutrophil percentage as influential predictors. For differentiating complicated versus uncomplicated appendicitis, SVC achieved moderate accuracy (70.19%) and ROC-AUC (76.33%), but low precision (14.85%) indicated challenges in minimizing false positives.

**Conclusion:**

Machine learning models based on CBC and CRP show preliminary potential for predicting appendicitis, but given the surgical-only cohort and modest performance, further validation is needed before clinical use, particularly in low-resource settings.

## Introduction

Acute appendicitis is a prevalent cause of acute abdominal pain. The lifetime risk of acquiring this medical condition is around 7%, but the probability of appendix perforation will go above 20% in the elderly [1,2]. Early and accurate diagnosis of appendicitis is crucial to prevent complications [3]. The mortality rate linked to appendicitis in the general population is under 1%, yet it may rise to 50% among elderly patients [4,5]. Delayed diagnosis of acute appendicitis may result in severe complications, including perforation and peritonitis. In pediatric populations, the condition frequently manifests at a later stage and with atypical symptoms, necessitating careful diagnostic approaches [6]. Appendectomy is the treatment of choice for acute appendicitis [7]. The incidence of negative appendectomies, defined as appendectomies performed without confirmed appendicitis, is notably high, reaching up to 50% in pediatric cases [8].The challenges emphasize the necessity for better diagnostic accuracy.

Efforts have been implemented to decrease the incidence of negative laparotomies in cases of suspected acute appendicitis. Despite comprehensive history taking, physical examination, and standard laboratory tests, reaching an accurate decision, especially in the initial phases of acute appendicitis, remains difficult. Recognized manifestations of acute appendicitis include right lower quadrant (RLQ) abdominal pain and elevated leukocyte count; however, these findings are not definitive on their own. Imaging techniques, especially ultrasonography and CT scans, are commonly employed and frequently aid in diagnosis and evaluation of complications [9–11]. A prospective study indicated that the accuracy of ultrasonography conducted by a skilled practitioner exceeded the initial clinical diagnosis made by a surgeon [12].

Various laboratory markers have been utilized to diagnose and assess the severity of appendicitis. White blood cell (WBC) counts generally rise in cases of appendicitis; however, they are not dependable indicators of severity or complications [13]. Complete Blood Cell parameters showed usefulness in diagnosing acute appendicitis specially in the area of machine learning utilization. C-reactive protein (CRP) serves as a diagnostic marker for various inflammatory conditions and has been identified as a valuable preoperative predictor of the severity of appendicitis [14–17].

Machine learning (ML), a subset of artificial intelligence (AI), has recently gained prominence in data analysis and has demonstrated efficacy in diagnosing acute appendicitis [16]. Machine learning distinguishes itself from conventional statistical methods by its ability to predict new observations through the analysis of existing data. Machine learning models employ large datasets to detect complex statistical patterns, exhibiting improved predictive performance compared to traditional approaches [18]. The efficacy of machine learning models is depending upon the quantity and quality of training data, alongside the appropriate calibration of model accuracy.

In recent years, studies have demonstrated promising results in the application of artificial intelligence and machine learning for predicting appendicitis [14–16,19–21]. However, the development of models utilizing a minimal number of variables—suitable for rapid, simple implementation in low-resource settings—has received comparatively less attention.

This study aims to develop a streamlined machine learning model based on demographic factors—specifically age and gender—and readily available laboratory markers, including complete blood count (CBC) parameters (white blood cell count, neutrophil count, lymphocyte count, and platelet count) as well as C-reactive protein (CRP), to support the diagnosis of acute appendicitis. The primary objective is to construct a model with acceptable diagnostic accuracy that can reduce the rate of negative appendectomies and distinguish between complicated and uncomplicated cases of appendicitis using these easily obtainable variables. This approach holds promise for improving clinical outcomes and reducing healthcare costs, particularly in resource-limited settings where access to advanced diagnostic modalities is restricted.

## Materials and methods

### Data collection

This retrospective, single-center study evaluated patients who underwent appendectomy at Besat Hospital in Hamadan for suspected acute appendicitis between January 2022 and December 2023. The study was approved by the research ethics committees of Hamadan University of Medical Sciences (Approval Number: IR.UMSHA.REC.1403.890), and the data were accessed for research purposes on 01/03/2025. Data collection was conducted anonymously after ethical approval, using the hospital’s electronic medical record system. The requirement for informed patient consent was waived by the Ethics Committee, as this was a retrospective study using anonymized data. Clinical data included patient age, gender, laboratory results (e.g., complete blood count with differential, C-reactive protein), and histopathological reports. No personal identifiers were accessed at any stage of the study; all data were de-identified prior to analysis to ensure patient confidentiality.

Patients were excluded if they had incomplete laboratory or pathology records, chronic medical conditions, elective or incidental appendectomy, or alternative diagnoses such as carcinoid tumors or parasitic infections.

Histopathological findings were categorized into three groups: (1) normal appendix (negative appendectomy), (2) uncomplicated appendicitis, characterized by transmural neutrophilic infiltration without evidence of gangrene or perforation, and (3) complicated appendicitis, defined by ischemic necrosis, gangrene, or perforation. Appendectomy specimens were classified based on detailed histopathological diagnoses. Complicated appendicitis included cases reported as Gangrenous Appendicitis (G) or Perforated Appendicitis. All other pathological categories—Acute Appendicitis (AA), Acute Appendicitis with Peri-appendicitis (AA PA), Acute Suppurative Appendicitis (ASA), Acute Suppurative Appendicitis with Peri-appendicitis (ASA PA), Fibrous Obliterated Appendix (FOA), Inflammatory Appendicitis with Reactive Lymphoid Follicular Hyperplasia (IA RLFH), Peri-appendicitis (PA), Peri-suppurative Appendicitis with Peri-appendicitis (PSA PA)—were classified as non-complicated appendicitis. Intact Vermiform Appendix (IVA) and other non-inflammatory findings were categorized as negative appendectomies. These pathological groups were used as outcome labels for model training and validation.

All laboratory tests were subject to quality control procedures, and histopathological interpretations were conducted by board-certified pathologists. Any discrepancies in data entry were resolved through secondary review by the clinical team. All personal identifiers were anonymized prior to analysis.

### Dataset description

This study was based on a clinical dataset comprising 453 anonymized records of patients assessed for suspected acute appendicitis. The dataset included demographic features such as age and gender, laboratory parameters including white blood cell count, neutrophil percentage, lymphocyte percentage, platelet count, and C-reactive protein, as well as diagnostic labels derived from surgical and pathological outcomes. Two non-predictive identifiers, namely patient ID and name, were excluded from the analysis to preserve anonymity and maintain the relevance of features for modeling purposes. To ensure data completeness, we restricted our dataset to individuals with both WBC counts and CRP records fully available in the pathology laboratory information system. As a result, no missing values were present in the dataset, and no imputation or data removal procedures were required.

A binary target variable, referred to as Appendicitis, was constructed based on the surgical classification of each case. Patients who underwent either non-complicated or complicated appendectomy were labeled as positive cases of appendicitis, whereas those diagnosed with a negative appendectomy were labeled as negative. This classification facilitated the development of a binary classification framework to distinguish between patients with and without confirmed appendicitis.

### Data preprocessing

Initial inspection revealed no missing values across any of the dataset features, thereby eliminating the need for imputation procedures. All categorical variables, including gender, pathology results, and surgical classification, were numerically encoded using label encoding. Standardization was then applied to all numerical features using the StandardScaler method from the scikit-learn library. This transformation ensured that each feature had a mean of zero and unit variance, thereby promoting stable convergence for models sensitive to feature scaling such as support vector machines and neural networks.

### Handling class imbalance

An imbalance was observed in the class distribution of the target variable, with one class representing a minority of the total records. To address this imbalance and reduce classification bias, an upsampling strategy was employed. The minority class in the training set was resampled using the resample method from scikit-learn, wherein minority samples were randomly duplicated until the class distributions were equalized. This approach enhanced the models’ ability to learn from underrepresented cases and improved generalization across both classes.

### Model development and training

A suite of seven machine learning algorithms was implemented to perform binary classification of appendicitis status. These included support vector classifier with a linear kernel and probability calibration, random forest classifier based on bagging of decision trees [22], AdaBoost classifier which iteratively adjusts weights of weak learners [23], k-nearest neighbors classifier which infers labels based on feature proximity [24], Bernoulli naïve Bayes classifier grounded in probabilistic modeling of binary features [25], multilayer perceptron classifier as a form of feedforward neural network [26], and the XGBoost classifier, a gradient boosting framework designed for scalability and regularization [27].

Model development followed an 80:20 train-test split with stratified sampling to preserve the original class proportions in both subsets. Data preprocessing steps, including feature scaling and class balancing, were encapsulated within pipelines to prevent information leakage. Each model was trained and evaluated independently using consistent experimental settings.

### Evaluation strategy

The predictive performance of all models was evaluated using stratified five-fold cross-validation to ensure that each fold retained the class distribution of the original dataset. A range of performance metrics was calculated to assess both discrimination and calibration. These included accuracy, F1-score, area under the receiver operating characteristic curve (ROC-AUC), Matthews correlation coefficient, Cohen’s kappa, balanced accuracy, log loss, average precision score, and Brier score loss. Confusion matrices were also generated to analyze the classification outcomes in terms of true positives, false positives, true negatives, and false negatives.

### Model Interpretability

To enhance transparency and support clinical relevance, model predictions were interpreted using SHapley Additive exPlanations (SHAP). SHAP provides a unified framework for interpreting complex models by assigning each input feature a contribution value toward the prediction, grounded in cooperative game theory. These values represent how much each feature contributes, positively or negatively, to the model’s output for a particular instance. In this study, SHAP values were calculated and visualized using summary plots, which allowed for both global feature importance analysis and local interpretability of individual predictions. The methodology adheres to the principles of local accuracy, missingness, and consistency, making it one of the most robust tools for model explanation in high-stakes domains such as medicine [28,29].

### Calibration procedure

Probability calibration of the Support Vector Classifier (SVC) was performed using the *CalibratedClassifierCV* module in scikit-learn. Calibration was carried out with isotonic regression, a non-parametric, piecewise-constant transformation of predicted probabilities. To reduce overfitting, a five-fold stratified cross-validation scheme was embedded within the calibration step; thus, the calibration model was trained only on the corresponding training folds and subsequently applied to the validation folds. The entire procedure was implemented through the custom calibrate_and_analyze_thresholds() function, which automated model fitting, calibration, and probability extraction for downstream evaluation.

Calibration performance was quantified by comparing the uncalibrated and calibrated models using two standard metrics. Expected Calibration Error (ECE) was computed over 10 uniform probability bins to assess absolute differences between predicted and empirical event frequencies, and the Brier Score was calculated to measure the mean squared difference between predicted probabilities and true binary outcomes. Lower values for both metrics were interpreted as indicating superior calibration performance. Calibration curves were generated comparing uncalibrated vs. calibrated predictions, displaying the relationship between mean predicted probability (x-axis) and fraction of positives (y-axis) across 10 bins. The calibrated model shows improved alignment with the diagonal (perfect calibration line).

### Regularized logistic regression baseline

As an interpretable reference model, we implemented an L2-regularized logistic regression classifier using the same predictor set as all other machine learning models. Model fitting was performed using the *lbfgs* optimizer with an L2 (Ridge) penalty and a regularization strength parameter of *C* = 1.0. The maximum number of training iterations was set to 1000, and model stochasticity was controlled by fixing the random state to 42.

To quantify model interpretability and feature-level contributions, regression coefficients and corresponding odds ratios were estimated together with 95% confidence intervals using nonparametric bootstrap resampling (1000 iterations). For each predictor, we report: (i) the raw logistic regression coefficient, (ii) its 95% bootstrap confidence interval, (iii) the exponentiated coefficient as the odds ratio (OR), (iv) the corresponding 95% confidence interval for the OR, and (v) the standardized coefficient, computed by multiplying the raw coefficient by the standard deviation of the corresponding feature to enable comparability across predictors with different units and scales.

### Operating threshold analysis

To evaluate classification performance across decision cutoffs, operating thresholds were systematically assessed over a probability range of 0.10 to 0.90 in increments of 0.05. For each threshold value, the following performance metrics were computed: sensitivity, specificity, precision, F1-score, and overall accuracy. Two complementary criteria were used to identify optimal operating points. The first aimed to maximize the F1-score, defined as the harmonic mean of precision and recall, thereby balancing false positives and false negatives. The second criterion enforced a minimum sensitivity of ≥90%, reflecting a clinically oriented constraint prioritizing the detection of true positive cases; among thresholds meeting this condition, the one yielding the highest specificity was selected.

### Model hyperparameters and software reproducibility

All hyperparameters used in model development are provided in a comprehensive supplementary table (S1 Table) to ensure full reproducibility. For the Support Vector Classifier (SVC), a linear kernel was used with *C* = 1.0, probability calibration enabled (probability = True), and a fixed random state of 42. The XGBoost classifier was implemented with a binary logistic objective and log-loss evaluation metric; default settings included max_depth = 6, learning_rate = 0.3, and n_estimators = 100, with random state set to 42. The Multi-Layer Perceptron (MLP) was configured with two hidden layers of sizes 1024 and 2048 neurons, a maximum of 1000 training iterations, random state 42, and default parameters (activation = relu, solver = adam, alpha = 0.0001). The Random Forest model used 20 estimators with default depth and split parameters and random state 42. The K-Nearest Neighbors classifier applied 15 neighbors with default uniform weighting and Minkowski distance. The AdaBoost classifier used 15 estimators with learning rate 1.0 and the SAMME.R algorithm. Gaussian Naive Bayes was included without additional tuning (var_smoothing = 1e-09).

### Model explainability using SHAP (SHapley Additive exPlanations)

Model interpretability was conducted using SHAP. We applied **LinearSHAP (LinearExplainer)**, which provides exact SHAP values for linear models and is appropriate for the linear Support Vector Classifier used in this study. SHAP values were computed using the **standardized training dataset** (n = 362) as the background distribution. All features were standardized using *StandardScaler* prior to model training, and all SHAP analyses reflect these standardized inputs. Global model explanations were generated using a **SHAP beeswarm plot**, and local explanations were produced using **force** and **waterfall** plots for a representative test case.

### Evaluation metric formulations

Let TP, TN, FP, and FN denote the number of true positives, true negatives, false positives, and false negatives, respectively. Let yᵢ denote the true label of the i-th sample, ŷᵢ its predicted class label, and pᵢ the predicted probability for the positive class. The evaluation metrics used in this study are defined as follows:


Accuracy = (TP + TN) / (TP + TN + FP + FN)

Precision = TP / (TP + FP)

Recall = TP / (TP + FN)

F1−score = 2TP / (2TP + FP + FN)

ROC−AUC = ∫ TPR(t) d(FPR(t)), where TPR = TP / (TP + FN), FPR = FP / (FP + TN)

MCC = (TP ×TN − FP ×FN) / sqrt((TP + FP)(TP + FN)(TN + FP)(TN + FN))

Kappa = (po − pe) / (1 − pe)

po = (TP + TN) / N

pe = ((TP + FP)(TP + FN) + (FN + TN)(FP + TN)) / N²

Balanced Accuracy = 0.5 × (TP / (TP + FN) + TN / (TN + FP))

$$\text{Brier}\;\text{Score}\;=\;(\text{1}\;/\;\text{N})\;\times \;\Sigma \;({\text{p}}_{\text{i}}\;-\;{\text{y}}_{\text{i}})²$$

$$\text{Log}\;\text{Loss}\;=\;-(\text{1}\;/\;\text{N})\;\times \;\Sigma \;[{\text{y}}_{\text{i}}\;\text{log}({\text{p}}_{\text{i}})\;+\;(\text{1}\;-\;{\text{y}}_{\text{i}})\;\text{log}(\text{1}\;-\;{\text{p}}_{\text{i}})]$$

### Computational environment, reproducibility, and code availability

All analyses were performed using Python (v3.13.2). The following package versions were used to ensure full reproducibility: pandas (2.2.3), numpy (2.1.0), scikit-learn (1.6.1), XGBoost (2.1.4), matplotlib (3.10.1), seaborn (0.13.2), SHAP (0.47.1), and scipy (1.15.2).

To maintain consistency across all modeling steps, a fixed random state (42) was applied to data splitting (train_test_split), upsampling procedures, shuffling, Stratified K-Fold cross-validation, and all machine learning algorithms requiring a random seed (Logistic Regression, SVC, Random Forest, XGBoost, AdaBoost, and MLP). Bootstrap sampling also used np.random.seed (42).

The full codebase, including preprocessing scripts, model training pipelines, and SHAP interpretability routines, is publicly accessible to support transparent replication of results (GitHub: https://github.com/mamishere).

## Results

### Demographic and clinical characteristics

Among the 453 patients included in the study, 312 (68.87%) were diagnosed with appendicitis, while 141 (31.13%) did not have appendicitis. Males accounted for 239 (52.76%) of the total sample, and females comprised 214 (47.24%). A significant association was observed between gender and appendicitis diagnosis (χ² = 15.53, p < 0.001), with a higher prevalence of appendicitis in males.

The mean age of patients with appendicitis was 26.71 years (SD = 15.95), which was significantly higher than the mean age of those without appendicitis (21.85 years, SD = 11.38) (p = 0.0012).

### Laboratory findings

Patients with appendicitis had significantly elevated white blood cell (WBC) counts compared to those without appendicitis (p < 0.001). Neutrophil levels were also higher in the appendicitis group compared to the non-appendicitis group (p < 0.001). Conversely, lymphocyte levels were significantly lower in patients with appendicitis compared to those without appendicitis (p < 0.001). There was no significant difference in platelet levels between the two groups (p = 0.4284). C-reactive protein (CRP) levels were significantly elevated in patients with appendicitis compared to the non-appendicitis group (p < 0.001). Table 1 summarizes patients characteristics.

**Table 1:** Patients characteristics.

Variable	Appendicitis (n = 312)	No Appendicitis (n = 141)	p-value
Gender (Male/Female)	190/ 122	49/ 92	< 0.001
Mean Age (years)	26.71 ± 15.95	21.85 ± 11.38	0.0012
WBC (cells/µL)	12,981.73 ± 4,089.13	10,332.62 ± 4,194.14	< 0.001
Neutrophils (%)	81.06 ± 9.87	72.65 ± 12.96	< 0.001
Lymphocytes (%)	16.47 ± 9.59	24.86 ± 12.36	< 0.001
Platelets (×10³/µL)	243839.7 ± 77537.63	249936.2 ± 71746.7	0.4284
CRP (mg/dL)	1.15 ± 1.19	0.65 ± 1.02	< 0.001

### Pathological classification

Among 312 patients diagnosed with appendicitis, various types were identified: AA in 88 cases (19.43%), Acute AA PA in 35 cases (7.73%), ASA in 43 cases (9.49%), and ASA PA in 118 cases (26.05%). Other types included FOA in 13 cases (2.87%), Gangrenous Appendicitis in 20 cases (4.42%), IA RLFH in 4 cases (0.88%), IVA in 128 cases (28.26%), PA and PSA PA in 1 case (0.22%) each, and Perforated Appendicitis in 2 cases (0.44%). Of the patients, 290 (64.02%) had non-complicated appendicitis, 22 (4.86%) had complicated appendicitis, and 141 (31.13%) had negative appendectomies.

### Performance of models

#### Phase 1: Classification of Positive vs. Negative Appendectomy.

A total of seven machine learning models were developed and evaluated for the classification of positive versus negative appendectomy cases. Performance was assessed using multiple evaluation metrics, including accuracy, precision, recall, F1-score, ROC AUC, PR AUC, specificity, balanced accuracy. The results are summarized in Tables 2 and 3.

**Table 2:** Phase 1 main performance of models in classification of positive vs. negative appendectomy, with corresponding confidence interval. Abbreviations: SVM = Support Vector Machine; KNN = K-Nearest Neighbors; MLP = Multi-Layer Perceptron; ROC AUC = Receiver Operating Characteristic Area Under the Curve; CI = Confidence Interval.

Model	ROC AUC	F1 Score	Accuracy	Specificity	Precision	Sensitivity (Recall)
**SVM**	76.39% (63.49–86.82)	82.54% (74.14–89.04)	75.82% (65.93–83.54)	60.71% (40.91–77.78)	82.54% (71.21–91.18)	82.54% (72.31–91.23)
**Random Forest**	72.82% (61.45–83.14)	78.52% (70.23–85.71)	68.13% (57.14–76.92)	32.14% (12.12–45.84)	73.61% (62.19–82.43)	84.13% (76.19–93.94)
**KNN**	70.92% (59.22–81.92)	75.00% (64.96–83.20)	67.03% (57.14–76.92)	57.14% (39.29–76.48)	78.95% (67.86–89.66)	71.43% (60.32–82.61)
**XGBoost**	72.85% (61.90–83.45)	82.01% (73.84–88.44)	72.53% (62.64–81.32)	32.14% (15.38–50.00)	75.00% (64.61–84.21)	90.48% (82.81–96.97)
**AdaBoost**	73.24% (61.43–83.37)	77.05% (67.89–84.55)	69.23% (59.34–78.02)	57.14% (38.71–76.00)	79.66% (68.42–90.00)	74.60% (63.64–84.48)
**Naive Bayes**	74.89% (62.90–86.21)	83.72% (76.80–90.08)	76.92% (69.23–85.71)	57.14% (40.00–75.86)	81.82% (72.30–90.48)	85.71% (76.27–93.55)
**MLP**	72.00% (61.18–82.54)	77.27% (68.29–85.11)	67.03% (57.14–76.92)	35.71% (18.18–53.85)	73.91% (62.71–83.82)	80.95% (70.49–90.74)

**Table 3:** Phase 1 additional performance metrics. Abbreviations: SVM = Support Vector Machine; KNN = K-Nearest Neighbors; MLP = Multi-Layer Perceptron; PR AUC = Precision–Recall Area Under the Curve; MCC = Matthews Correlation Coefficient; G-Mean = Geometric Mean; SVM = Support Vector Machine; KNN = K-Nearest Neighbors; MLP = Multi-Layer Perceptron.

Model	PR AUC (%)	Balanced Accuracy (%)	Log Loss	Brier Score	MCC	Cohen’s Kappa	G-Mean (%)
**SVM**	83.01	71.63	0.5831	0.1879	0.4325	0.4325	70.79
**Random Forest**	84.62	58.13	0.9235	0.1937	0.1848	0.1786	52
**KNN**	82.09	64.29	1.3241	0.2077	0.2726	0.2697	63.89
**XGBoost**	85.9	61.31	0.8755	0.2287	0.2814	0.2597	53.93
**AdaBoost**	83.33	65.87	0.58	0.1965	0.3069	0.3053	65.29
**Naive Bayes**	81.6	71.43	0.8399	0.1784	0.4431	0.4417	69.99
**MLP**	85.85	58.33	2.4133	0.2852	0.1797	0.1772	53.77

**Fig 1:**
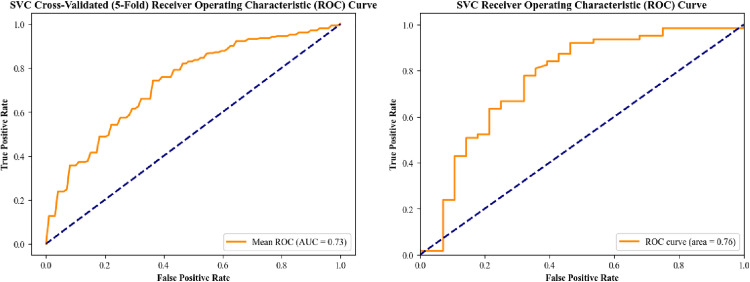
SVC Receiver Operating Characteristic (ROC) Curves for phase 1: With and Without 5-Fold Cross-Validation.

Among all models, the Support Vector Classifier (SVC) demonstrated the highest overall performance, achieving an accuracy of 75.82%, precision and recall of 82.54%, F1-score of 82.54%, and a ROC AUC of 76.39% (Fig 1). It also exhibited the best PR AUC (83.01%) and balanced accuracy (71.63%), with a G-Mean of 70.79%.

**Fig 2:**
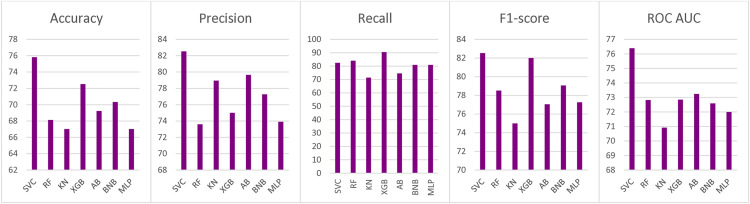
Comparison of Model Performance Metrics: F1 Score, Accuracy, AUC, Precision, and Recall.

Other models such as XGBoost and GaussianNaive Bayes also showed competitive performance. XGBoost achieved a recall of 90.48% with an F1-score of 82.01%, while GaussianNB reached an F1-score of 83.72% with balanced performance across most metrics. The Random Forest Classifier, despite high recall (84.13%), demonstrated low specificity (32.14%) and a comparatively lower balanced accuracy (58.13%). A comparison of the models across selected metrics is shown in Fig 2.

Cross-validation analysis for the SVC model provided further insights into its robustness. The average confusion matrix revealed 44 true positives and 18 true negatives, with 10 false positives and 19 false negatives. The cross-validation performance metrics included an average ROC AUC of 73.32%, F1-score of 74.93%, accuracy of 67.77%, and balanced accuracy of 66.55%. The precision and recall were 80.89% and 69.87%, respectively. Additional metrics like MCC (31.18%) and Cohen’s Kappa (30.42%) further supported the reliability of this model.

### Feature importance analysis

**Fig 3:**
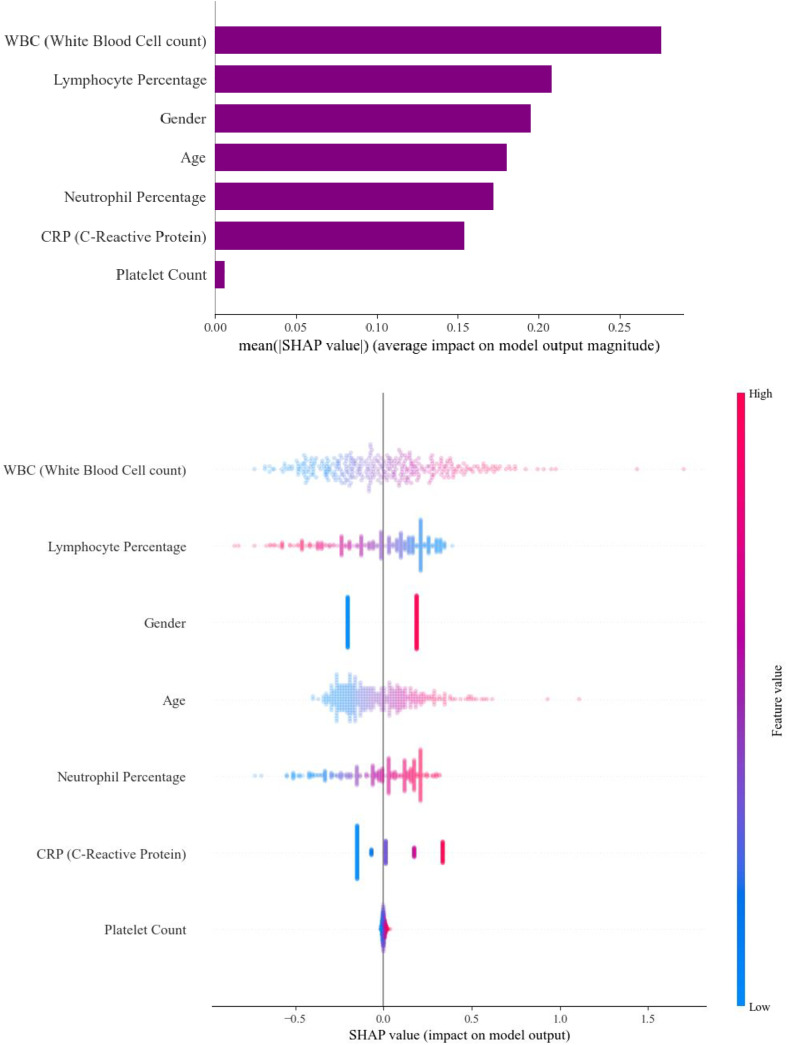
SHAP Value Plots: Individual Impact and Mean Impact on Model Output.

SHAP values were used to interpret the SVC model., the most influential features in determining the classification were white blood cell count (WBC) with an average SHAP value of 0.276, followed by lymphocyte percentage (0.208), gender (0.195), age (0.180), and neutrophil percentage (0.172). C-reactive protein (CRP) and platelet count had relatively lower contributions (Fig 3).

A SHAP **beeswarm plot** demonstrated consistent global feature influence patterns across the cohort. Local interpretability was assessed using force and waterfall plots for a representative test case (predicted probability 0.625), illustrating how individual standardized feature values shifted model output relative to the baseline prediction (S1 and S2 Figs).

### Regularized Logistic Regression Results (Phase I)

The L2-regularized logistic regression model demonstrated statistically significant associations for several predictors. Higher white blood cell count (WBC) was associated with increased odds of appendicitis (OR = 1.93; 95% CI: 1.42–2.75), while lower lymphocyte percentage was inversely associated with appendicitis risk (OR = 0.44; 95% CI: 0.23–0.90). Gender was also a significant predictor, with an odds ratio of 1.49 (95% CI: 1.24–1.82), indicating a measurable association with disease likelihood. Significance was defined as a 95% confidence interval for the odds ratio not crossing 1.0.

Model performance assessment showed a ROC AUC of 78.12% (95% CI: 66.02–88.22%) and an F1-score of 81.60% (95% CI: 73.60–88.37%). The model achieved an overall accuracy of 74.73% (95% CI: 64.84–83.52%) with a sensitivity of 80.95% (95% CI: 70.31–90.28%) and specificity of 60.71% (95% CI: 42.84–77.78%). Precision was 82.26% (95% CI: 72.06–90.91%), and the PR AUC was 84.16% (95% CI: 73.18–95.16%). Additional metrics included a balanced accuracy of 70.83% (95% CI: 60.06–80.32%), Matthews correlation coefficient of 0.4127 (95% CI: 0.2015–0.6010), and Cohen’s κ of 0.4126 (95% CI: 0.1973–0.6008). The geometric mean (G-Mean) was 70.11%. Probabilistic performance measures showed a log loss of 0.5684 and a Brier Score of 0.1805, indicating moderate calibration and prediction reliability. Supplementary table (S1 Table) includes features coefficients.

### Phase 2: Classification of Complicated vs. Uncomplicated Appendicitis Among Positive Cases

In the second phase of the study, the aim was to classify complicated versus uncomplicated appendicitis among patients with confirmed (positive) appendectomy. The Support Vector Classifier (SVC) was employed for this binary classification task. This phase faced a sever class imbalance so results are mostly exploratory and underpowered.

**Fig 4:**
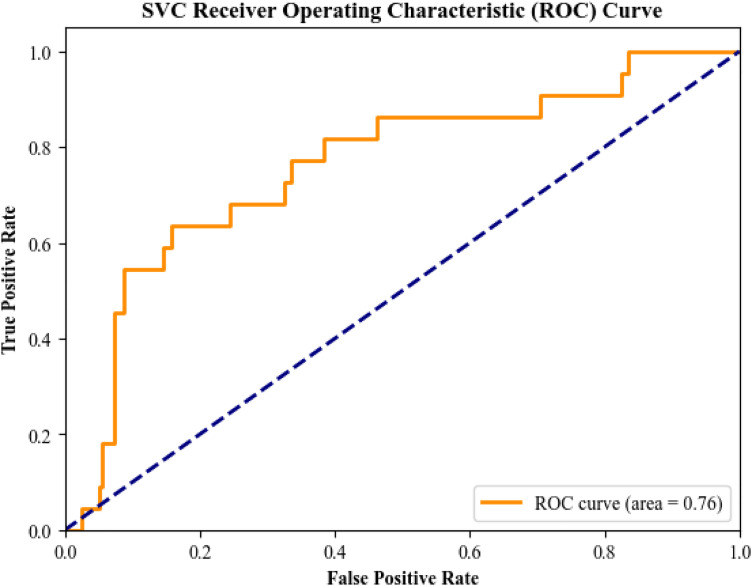
SVC Receiver Operating Characteristic (ROC) Curves for phase 2.

The SVC model achieved an accuracy of 70.19% and a ROC AUC of 76.33% (Fig 4), indicating reasonable discriminative ability. The balanced accuracy was 69.26%, and the G-Mean reached 69.25%, reflecting a relatively balanced performance between sensitivity and specificity.

However, the precision was notably low (14.85%), despite a moderate recall of 68.18%, resulting in a F1-score of 24.39%. This suggests that while the model was able to detect a fair number of true complicated cases, it struggled with a high false-positive rate. The specificity was 70.34%, showing moderate ability to identify uncomplicated cases correctly.

Other evaluation metrics included a PR AUC of 19.31%, log loss of 59.62, and Brier score of 19.93. The Matthews correlation coefficient (MCC) and Cohen’s kappa were 21.08% and 14.49%, respectively, indicating limited agreement between predicted and actual labels.

These results suggest that while the SVC model shows potential in distinguishing complicated from uncomplicated appendicitis, especially in terms of ROC AUC and recall, further optimization or alternative models may be necessary to improve precision and overall predictive balance.

### Phase I calibration and threshold analysis

**Fig 5:**
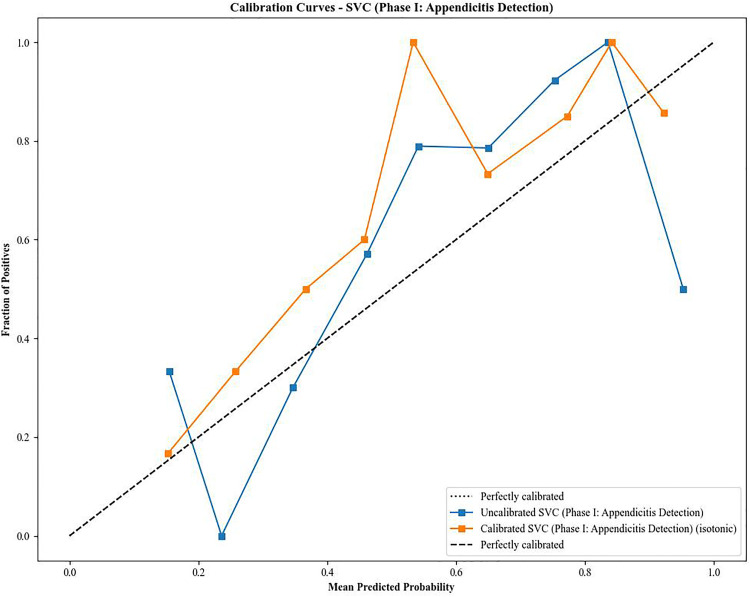
Calibration Curves for Phase I Appendicitis Detection Model. Calibration plot comparing uncalibrated and isotonic-calibrated probability estimates. The x-axis represents predicted probabilities, and the y-axis reflects observed outcome frequencies. The calibrated model demonstrates improved alignment with the diagonal reference line, indicating enhanced probability reliability and reduced Expected Calibration Error (ECE).

Calibration analysis demonstrated substantial improvement in predicted probability reliability following isotonic regression (Fig 5). The uncalibrated model yielded an Expected Calibration Error (ECE) of 0.1723 and a Brier Score of 0.1879. After applying isotonic calibration with five-fold cross-validation, the ECE decreased to 0.1007, representing a 41.6% relative reduction, while the Brier Score improved to 0.1773 (5.6% relative reduction).

**Fig 6:**
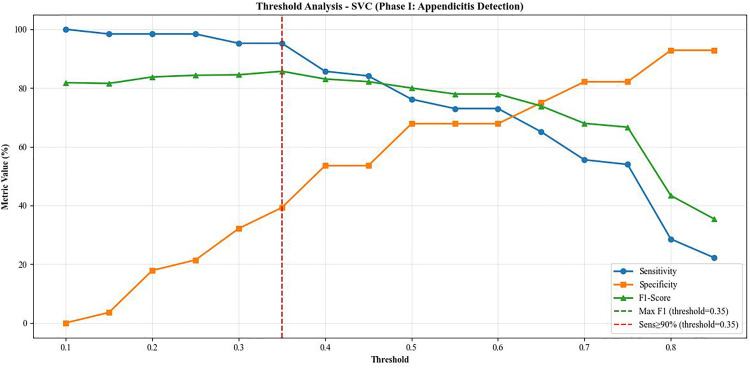
Operating Threshold Performance Across Probability Cutoffs. Threshold analysis showing sensitivity, specificity, precision, F1-score, and accuracy across probability thresholds ranging from 0.10 to 0.90 (step = 0.05). The optimal clinical operating point (threshold = 0.35) satisfies both predefined criteria: maximum F1-score and minimum sensitivity ≥90%, achieving a balanced trade-off between case detection and false-positive rate.

Operating threshold analysis identified an optimal decision cutoff at 0.35 under both predefined criteria. When maximizing the F1-score, the threshold of 0.35 achieved a sensitivity of 95.24%, specificity of 39.29%, precision of 77.92%, F1-score of 85.71%, and overall accuracy of 78.02%. Applying the clinically oriented criterion (Fig 6) requiring sensitivity ≥90% led to convergence on the same threshold value. Thus, at a 0.35 decision cutoff, the model satisfies the high-sensitivity requirement while simultaneously yielding the highest F1-score among candidate thresholds, indicating a favorable balance between case detection and false-positive burden in the appendicitis detection setting.

## Discussion

This retrospective study assessed the application of machine learning (ML) models for diagnosing acute appendicitis and differentiating between complicated and uncomplicated cases utilizing solely simple laboratory and demographic data. The objective was to create a therapeutically beneficial decision-support model with minimum input features. Our models indicated that a limited set of commonly accessible markers—white blood cell count, neutrophil count, lymphocyte count, platelet count, and CRP—can achieve satisfactory ability to diagnose for acute appendicitis. This minimalistic approach is especially helpful in low-resource settings where access to imaging or extensive laboratory testing may be limited. Although we believe that a combination of clinical and imaging data will enhance accuracy, our results show the potential of an effective, accessible model for early triage and risk evaluation.

In differentiating patients with appendicitis (those having positive appendectomy findings) from those without (negative appendectomy), the most effective support vector classifier reached an accuracy of 75.82%, as well as a high F1-score of 82.54% and a ROC AUC of 76.39%. This means that approximately three-quarters of cases were accurately classified based only on these inflammatory markers. The strong F1-score, which addresses precision and recall, indicates that the model effectively balanced the avoidance of false negatives with the reducing of false positives. To avoid overestimating performance from a single split, we emphasized the 5-fold cross-validation results, which showed more conservative but stable estimates (ROC-AUC 73.3%, F1-score 74.9%, precision 80.9%, recall 69.9%). In terms of classifying severity (complicated versus uncomplicated appendicitis), the model’s performance was comparatively modest. It provided an acceptable recall in detecting complicated cases but at the price of lesser accuracy – in reality this means the algorithm successfully captured most actually complicated patients but also highlighted some uncomplicated cases as possibly severe. Clinically, this pattern (high sensitivity with moderate specificity) may be seen acceptable when the objective is to prevent the missing a perforated appendicitis, however it may lead to some over-triage. Attaining an AUC of approximately 76% solely from standard laboratory tests is promising, highlighting that a minimally dependent model can aid in the diagnosis of appendicitis in the absence of clinical or imaging data. The results indicate that our model, although not flawless, may function as a screening tool: it succeeds in confirming appendicitis (with an F1-score exceeding 80%) and offers a conservative yet reasonable attempt to identify complicated cases.

Although performance varies on input characteristics and research design, our results match a growing body of data endorsing the effectiveness of machine learning in appendicitis diagnosis. Unlike Akmese et al. (2020) [16], who reported over 95% accuracy using gradient boosting with a more extensive clinical dataset, our minimalistic model compromises some accuracy for simplicity. Similarly, Mijwil and Aggarwal (2022) [15] obtained roughly 84% accuracy using simple criteria and a balanced dataset, implying that, given limited data, good predictive performance is possible even. The inclusion of extra demographic or routine laboratory variables may help to explain their slightly better performance in their study; nevertheless, the results highlight the importance of fundamental characteristics in appendicitis prediction overall. In comparison to studies using just laboratory models, our study theoretically aligns Akbulut et al. (2023) [14], who achieved an accuracy of 88.2% by using a larger and more diverse set of laboratory markers including bilirubin. Their better performance suggests that even a small increase of our feature set could improve accuracy while maintaining the low-resource applicability we aim for.

Pediatric studies provide useful similarities: Reismann et al. (2019) [21] reported significant performance drops in identifying complicated appendicitis despite using both labs and ultrasound to reach 90% diagnostic accuracy, which is consistent with our own findings; Marcinkevics et al. (2021)[30] showed that a small set of high-impact features could replicate results of models using dozens of variables. These findings support a key finding of our study: that, regardless of patient age or dataset richness, appendicitis diagnosis is a more achievable machine learning problem than severity differentiation.

Recent studies have also addressed models with extensive feature inclusion and highlighted the importance of interpretability in machine learning-based tools. For example, Chadaga et al. (2024) [31] achieved over 94% accuracy by utilizing Xgboost algorithm and a wide range of variables, including laboratory data, imaging findings, the Alvarado scoring system, clinical symptoms, and even the length of hospital stay. However, despite its high value, their method lacks practical usefulness due to its complexity. Using merely CBC and CRP, our work indicates what is practical in areas where access to imaging or large labs is restricted. Previous studies and our own support a basic theme: machine learning may enhance appendicitis diagnosis; model performance is highly influenced by the volume and kind of data used. Our concept provides a low-barrier, logical approach perfect for triaging in low-resource or emergency settings, therefore helping particularly in this field. Although it is not a replacement for thorough research, supported by convergent findings throughout the literature it offers a reasonable diagnosis when clinical or imaging data are absent.

A meta-analysis of 2,161 patients [32] reported that the RIPASA score achieved a sensitivity of 94%, specificity of 55%, and an AUC of 0.94, while the Alvarado score showed 69% sensitivity, 77% specificity, and an AUC of 0.79. In comparison, our machine learning model reached an AUC of 76.4% and an F1-score of 82.5%. These findings suggest that our model performs at a level comparable to the Alvarado score in terms of overall discriminative ability, while offering stronger balance between precision and recall. Although RIPASA demonstrated higher AUC, its lower specificity highlights the trade-off between sensitivity and false positives, underscoring the potential of machine learning approaches to provide a more balanced diagnostic aid.

The results of this single-center, retrospective study might not be very generalizable. Using just age, CBC, and CRP values, the model was developed excluding clinical and imaging data that might improve accuracy. There was no external validation, thus real-world applicability is still unknown. Another limitation is the very small number of complicated appendicitis cases (n = 22), which likely contributed to the model’s low precision in this subgroup. Larger, multicenter datasets will be needed to improve predictive accuracy and generalizability.

Future research should prioritize prospective multicenter validation to ascertain the model’s generalizability across varied clinical environments. Including fundamental clinical indicators or supplementary low-cost laboratory markers may improve predictive precision while maintaining accessibility. Comparative research against traditional scoring systems might explain the advantages of machine learning in resource-limited settings. Ultimately, the objective is to create a straightforward, expandable tool—such as a mobile application—that frontline practitioners can utilize to help with real-time decision-making and minimize unnecessary surgical interventions.

## Conclusion

Our study demonstrates that machine learning models using only routine lab markers like CBC and CRP can reasonably predict both acute and complicated appendicitis. While not a replacement for comprehensive evaluation, this minimalist approach offers a practical diagnostic aid in low-resource or emergency settings. With further validation and thoughtful enhancement, such models have strong potential to improve early decision-making and reduce unnecessary interventions.

## Supporting information

S1 Fig SHAP force plot for an individual prediction. Feature contributions to the SVC model output.
(JFIF)

S2 Fig SHAP waterfall plot for an individual prediction. Cumulative feature effects on the SVC model prediction.
(JFIF)

S1 Table Logistic regression results for Phase I appendicitis detection. Coefficients, odds ratios, and 95% confidence intervals.
(DOCX)

S1 File TRIPOD checklist for prediction model development. Completed TRIPOD reporting checklist.
(DOCX)
