# Supplementary figures and images for: Machine learning-based prediction of acute and complicated appendicitis using readily available data in low-resource settings
Source: PLoS One. 2026 Feb 3;21(2):e0339299. doi: 10.1371/journal.pone.0339299 (PMC12867214; doi:10.1371/journal.pone.0339299)

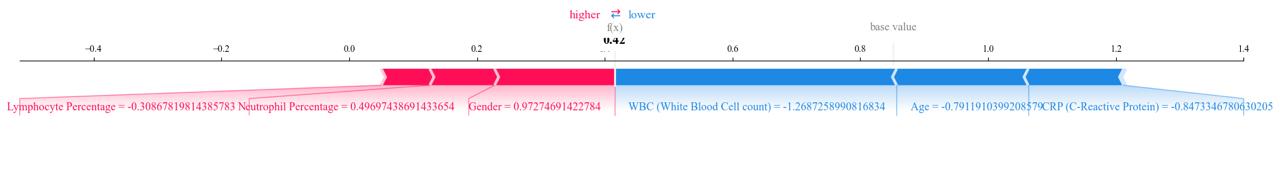

Supplement: S1 Fig — Feature contributions to the SVC model output. (JFIF) [file pone.0339299.s001.jfif]

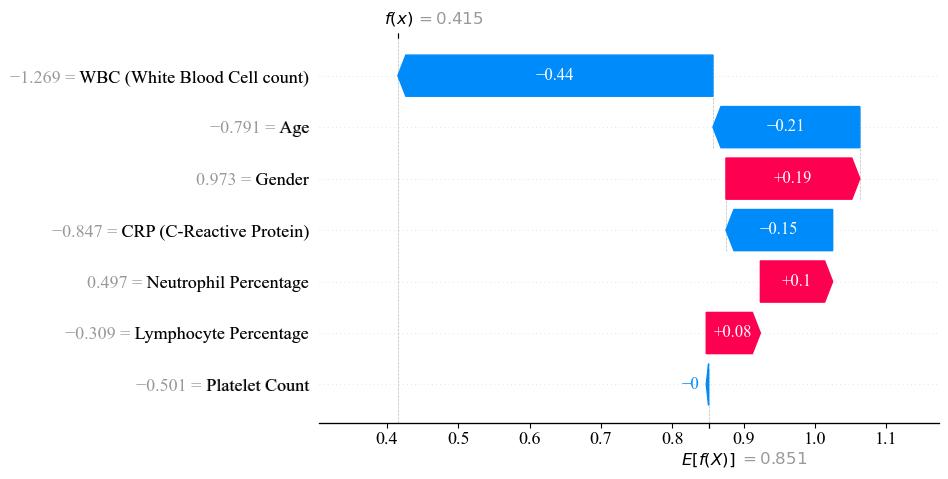

Supplement: S2 Fig — Cumulative feature effects on the SVC model prediction. (JFIF) [file pone.0339299.s002.jfif]
