# Supplementary material for: Machine learning-based prediction of acute and complicated appendicitis using readily available data in low-resource settings
Source: PLoS One. 2026 Feb 3;21(2):e0339299. doi: 10.1371/journal.pone.0339299 (PMC12867214; doi:10.1371/journal.pone.0339299)
Supplement: S1 Table — Coefficients, odds ratios, and 95% confidence intervals. (DOCX) [file pone.0339299.s003.docx]

Supp Table: **Logistic regression coefficients, odds ratios, and 95% confidence intervals for Phase I appendicitis detection model.**
Coefficients and corresponding odds ratios were estimated using L2-regularized logistic regression with 1000-iteration bootstrap resampling. Standardized coefficients represent the effect size after scaling by feature standard deviation. Features with 95% confidence intervals for the odds ratio not crossing 1.0 were considered statistically significant predictors.

| **Feature** | **Coefficient** | **Coefficient CI Lower** | **Coefficient CI Upper** | **Odds Ratio** | **OR CI Lower** | **OR CI Upper** | **Standardized Coefficient** |
| --- | --- | --- | --- | --- | --- | --- | --- |
| Age | 0.2901 | 0.0654 | 0.5152 | 1.3366 | 1.0676 | 1.6740 | 0.2904 |
| Gender | 0.3958 | 0.2121 | 0.5986 | 1.4856 | 1.2363 | 1.8195 | 0.3962 |
| WBC (White Blood Cell Count) | 0.6551 | 0.3522 | 1.0112 | 1.9253 | 1.4222 | 2.7489 | 0.6557 |
| Neutrophil Percentage | -0.5662 | -1.1925 | 0.1113 | 0.5677 | 0.3035 | 1.1177 | -0.5668 |
| Lymphocyte Percentage | -0.8120 | -1.4572 | -0.1040 | 0.4440 | 0.2329 | 0.9012 | -0.8128 |
| Platelet Count | 0.0652 | -0.1861 | 0.2899 | 1.0674 | 0.8302 | 1.3362 | 0.0652 |
| CRP (C-Reactive Protein) | 0.1704 | -0.0448 | 0.3760 | 1.1858 | 0.9562 | 1.4565 | 0.1706 |
